# P-653. Epidemiology of *Streptococcus pneumoniae* serotypes using serotype specific urinary antigen (SSUAD) in cancer patients

**DOI:** 10.1093/ofid/ofae631.850

**Published:** 2025-01-29

**Authors:** Melvili Cintron, Varshini Gali, Krupa Jani, Anna Kaltsas, Mini Kamboj, Susan K Seo, Genovefa Papanicolaou, Yeon Joo Lee

**Affiliations:** Memorial Sloan Kettering Cancer Center, New York, New York; Weill Cornell Medicine, New York, New York; Memorial Sloan Kettering Cancer Center, New York, New York; Memorial Sloan Kettering Cancer Center, New York, New York; MSKCC, New York, NY; Memorial Sloan Kettering, New York, New York; Memorial Sloan Kettering Cancer Center, New York, New York; Memorial Sloan Kettering Cancer Center, New York, New York

## Abstract

**Background:**

Cancer patients have a 2.4-fold higher risk of dying from pneumonia compared to the general population. *Streptococcus pneumoniae* is one of the most common etiologies of community-acquired pneumonia. Serotype specific information of nonbacteremic pneumococcal pneumonia in oncology settings is limited. Thus, we described the frequency of *S. pneumoniae* serotypes detected by a serotype-specific urinary antigen (SSUAD) assay in cancer patients.

**Table 1.**
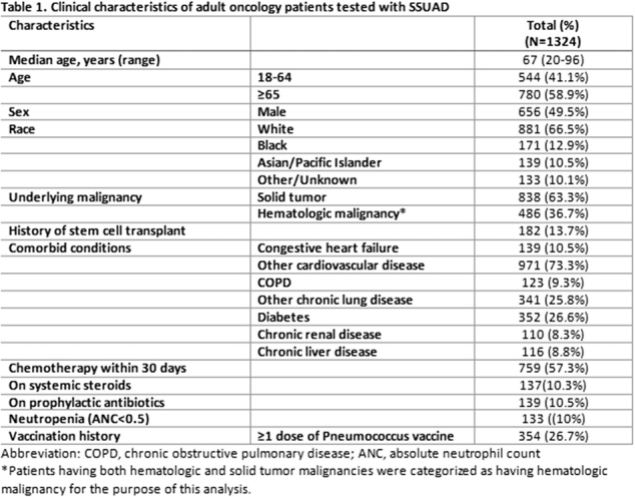
Clinical characteristics of adult oncology patients tested with SSUAD

**Methods:**

This was an interim analysis of a prospective observational study from 1/1/2023 - 1/31/2024. Cancer patients aged ≥18 years at MSKCC, inpatient or outpatient, with a *Streptococcus* urine antigen test (SUA) ordered at clinician’s discretion for diagnostic work-up of pneumonia were included. SUA was performed in-house using the BinaxNOW™, a lateral flow immunochromatographic test that detects C-polysaccharide cell wall protein common to all S. pneumoniae serotypes. Batched urine samples were tested using SSUAD, which detects the 15 pneumococcal serotypes (1, 3, 4, 5, 6A, 6B, 7F, 9V, 14, 18C, 19A, 19F, 22F, 23F, 33F) covered by 15-valent pneumococcal conjugate vaccine (PCV15) at Q2 laboratories (Quebec, Canada).

**Table 2.**
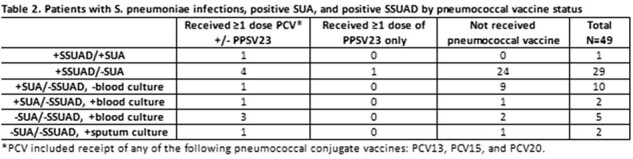
Patients with S. pneumoniae infections, positive SUA, and positive SSUAD by pneumococcal vaccine status

**Results:**

Of 1324 patients, 780 (58.9%) were ≥65 years old and 486 (36.7%) had hematologic malignancies (Table 1). Thirteen patients had positive SUA (1 positive SSUAD and 12 negative SSUAD). Thirty patients had a positive SSUAD and only 1 of these patients also had a positive SUA (Figure 1). In 9 patients who had *S. pneumoniae* recovered from a culture (sputum [N=2] or blood [N=7]), SUA was only positive in 2 of the blood culture positive patients and none by SSUAD. Among the 30 patients with positive SSUAD, pneumococcus serotype 7F was most common with 26.3%, followed by 3 (17.5%), 5 (7.0%), 18C (7.0%), and 33F (7.0%); 12 (40%) had ≥2 serotypes detected (Figure 2). Pneumococcal vaccination status of the 49 patients with corresponding SUA and SSUAD results are summarized in Table 2.

**Figure:**
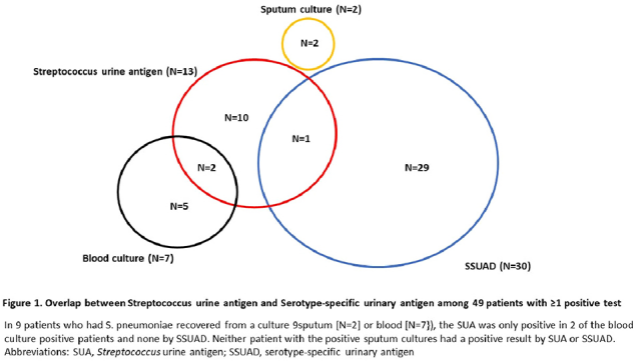

**Conclusion:**

**Among** cancer **patients with positive SSUAD detecting PCV15 serotypes, 7F (26.3%), 3 (17.5%), 5 (7.0%), 18C (7.0%), and 33F (7.0%) predominated.** More studies are needed to further understand the sero-epidemiology of circulating pneumococcal serotypes in this patient population.

**Figure:**
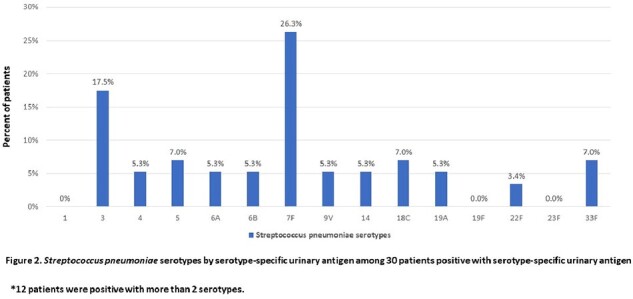

**Disclosures:**

**Melvili Cintron, PhD, D(ABMM)**, Copan Diagnostics: Grant/Research Support|Merck & Co: Grant/Research Support|Roche Diagnostics: Advisor/Consultant **Susan K. Seo, MD**, Merck: Grant/Research Support **Genovefa Papanicolaou, MD**, AlloVir: Advisor/Consultant|AlloVir: Data safety monitoring committee|Merck: Advisor/Consultant|Merck: Grant/Research Support|Merck: Investigator|Symbio: Advisor/Consultant **Yeon Joo Lee, MD, MPH**, AiCuris: institutional research support for clinical trials|Karius: institutional research support for clinical trials|Merck: Grant/Research Support|Scynexis: institutional research support for clinical trials

